# Nanotechnological engineering of extracellular vesicles for the development of actively targeted hybrid nanodevices

**DOI:** 10.1186/s13578-022-00784-9

**Published:** 2022-05-14

**Authors:** Bianca Dumontel, Francesca Susa, Tania Limongi, Veronica Vighetto, Doriana Debellis, Marta Canta, Valentina Cauda

**Affiliations:** 1 Department of Applied Science and Technology, Politecnico di Torino, Turin, Italy; 2 Electron Microscopy Facility, Istituto Italiano di Tecnologia (IIT), Genoa, Italy

**Keywords:** Extracellular vesicles, Zinc oxide nanocrystals, Targeting antibodies, Ultrasound shock waves, Bioengineering

## Abstract

**Background:**

We propose an efficient method to modify B-cell derived EVs by loading them with a nanotherapeutic stimuli-responsive cargo and equipping them with antibodies for efficient targeting of lymphoma cells.

**Results:**

The post-isolation engineering of the EVs is accomplished by a freeze–thaw method to load therapeutically-active zinc oxide nanocrystals (ZnO NCs), obtaining the so-called TrojanNanoHorse (TNH) to recall the biomimetism and cytotoxic potential of this novel nanoconstruct. TNHs are further modified at their surface with anti-CD20 monoclonal antibodies (TNH^CD20^) achieving specific targeting against lymphoid cancer cell line. The in vitro characterization is carried out on CD20+ lymphoid Daudi cell line, CD20-negative cancerous myeloid cells (HL60) and the healthy counterpart (B lymphocytes). The TNH shows nanosized structure, high colloidal stability, even over time, and good hemocompatibility. The in vitro characterization shows the high biocompatibility, targeting specificity and cytotoxic capability. Importantly, the selectivity of TNH^CD20^ demonstrates significantly higher interaction towards the target lymphoid Daudi cell line compared to the CD20-negative cancerous myeloid cells (HL60) and the healthy counterpart (lymphocytes). An enhanced cytotoxicity directed against Daudi cancer cells is demonstrated after the TNH^CD20^ activation with high-energy ultrasound shock-waves (SW).

**Conclusion:**

This work demonstrates the efficient re-engineering of EVs, derived from healthy cells, with inorganic nanoparticles and monoclonal antibodies. The obtained hybrid nanoconstructs can be on-demand activated by an external stimulation, here acoustic pressure waves, to exploit a cytotoxic effect conveyed by the ZnO NCs cargo against selected cancer cells.

**Graphical Abstract:**

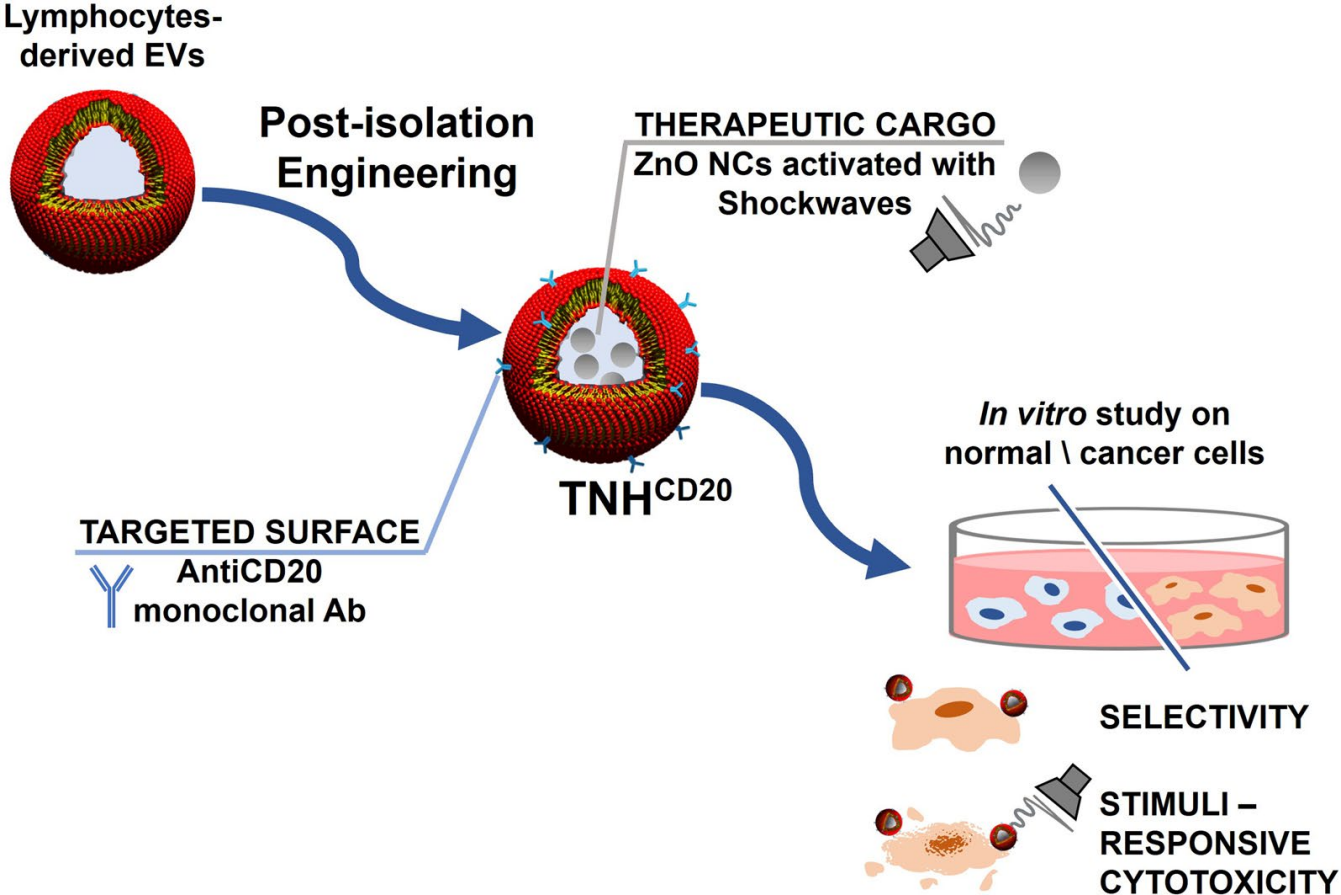

**Supplementary Information:**

The online version contains supplementary material available at 10.1186/s13578-022-00784-9.

## Background

In the last decades, an increasing number of studies allowed to unravel the biological role of extracellular vesicles (EVs), an heterogenous group of membrane-enclosed micro- and nano-structures secreted by different cell types, and to highlight their primary role in cell-to-cell communication. Starting from 2006, several groups reported that EVs could contain and transfer biomolecules present in the cytosol of originating cells [1], including mRNA and miRNA [2], proteins, lipids and metabolites [3]. The bioactive materials are protected by the vesicles from the extracellular environment and shuttled to neighboring or considerably distant cells, triggering functional changes in the recipient cells and regulating many physiological and pathological processes [4]. The discovery of their role of intercellular messengers, made EVs compelling candidates for the development of innovative therapeutic tools. Indeed, by opportunely customize their membranes and cargo, EVs can be engineered as highly biocompatible and specific cell-derived delivery tools.

Considering their lipid-bilayer structure, EVs can be assimilated to liposomes, i.e. synthetic lipid vesicles widely applied for the delivery of drugs [5], proteins [6] and inorganic nanoparticles (NPs) [7, 8]. As known, the lipid bilayer could provide a suitable defensive barrier to preserve the colloidal and chemical stability of different materials in the biological environment allowing the loading of either hydrophilic and hydrophobic compounds, stable in the vesicles core or in the lipid membrane, respectively [9]. However, with respect to synthetic liposomes, EVs would possess additional features like lower immunogenicity, prolonged blood-circulation and higher biocompatibility [10], as well as an intrinsic ability to cross different biological barriers [11].

Thanks to these promising features, EVs have been recently evaluated as efficient carriers for the delivery of therapeutic cargos like small molecule drugs, nucleic acids, genes and even NPs for the treatment of various pathologies, including neurodegenerative and cardiovascular diseases, inflammation, diabetes [4] and especially cancer. According to a query done on July 2021 on the US-NIH clinical trials database, 324 clinical trials on EVs or exosomes are currently registered, 121 of which refer to their use for both diagnostic and therapeutic oncological purposes. Despite EVs loaded with anticancer drugs [12–14], recent researches evaluated the combination of cell-derived vesicles with different types of inorganic NPs with the purpose to combine the stabilizing and biomimetic EVs’ features with the imaging [15, 16], drug delivery [17, 18] and/or therapeutic capabilities of the NPs [19].

However, the efficient encapsulation of external cargos and the maintenance of EVs integrity and functionalities after the loading processes are still the major challenges in their application as drug delivery systems [20]. Essentially, the current loading strategies can be divided in endogenous and exogenous methods, which involve respectively the engineering of parent cells for the production of pre-loaded vesicles or the direct engineering of EVs after their isolation. According to the modalities of combination between EVs and external payloads, post-isolation methods are conventionally divided into two further subcategories, i.e. passive or active loading methods [21]. In case of passive loading, the two components would simply interact on the basis of their physicochemical properties, exploiting, for instance, the presence of concentration gradients or the hydrophobicity of payloads to efficiently cross the EVs lipid membrane [9]. Although passive strategy is generally characterized by a good preservation of EVs membrane integrity, this is often accompanied by low encapsulation efficiency in the case of large or hydrophilic payloads, that cannot easily diffuse through lipid bilayers [22]. To overcome this issue, different active methods, which involve the application of electrical, mechanical or chemical external stimuli to destabilize EVs membrane and facilitate the cargos entrance, are widely investigated [23, 24]. Additionally, the engineering of EVs for drug delivery purposes is not limited to cargo customization but concerns also the biological and/or biochemical functionalization of their external surface, mainly devoted to improve their homing and target specificity. Although EVs harbor native homing and targeting properties [25], this intrinsic tropism is often insufficient to achieve an effective delivery within specific organ, tissues or cells. Concerning EVs application in oncology, single domain and monoclonal antibodies, small peptides and glycans [26] are currently investigated as ligands for biomolecular markers specific of the target cells and tumor microenvironment [10]. Furthermore, the mentioned intrinsic targeting ability and organotropism are mainly shown by tumor-derived EVs that, thanks to the presence of particular proteins and lipids in their membrane, are able to selectively home their parent tumors [27]. However, some literature studies highlighted that tumor-derived EVs are directly involved in the progression of cancer [28], metastasis promotion [29] and in the development of drug resistance phenomena [30]. Thus, their application as delivery platforms is not free of concerns and less hazardous alternatives, such as EVs derived from fruits [31], bovine milk [32] or healthy cells [33, 34], are intensively investigated.

In this scenario, the present study focuses on the post-isolation engineering of lymphocytes-derived EVs, evaluating their surface modification with anti-CD20 monoclonal antibodies and their loading with therapeutically active zinc oxide nanocrystals (ZnO NCs). The design, construction and characterization of a hybrid nanoconstruct, named Trojan nano-horse (TNH) to convey the concept of its biomimetism and cytotoxic potential, were conducted along with the evaluation of its biocompatibility, specificity and targeted cytotoxic capability. In particular, in vitro studies were performed on a CD20+ human lymphoid cancer cell line (Daudi), compared to healthy CD20+ lymphocytes and CD20− human myeloid neoplastic cell line (HL60). Moreover, the therapeutic activity of the targeted TNH^CD20^ was exploited in combination with high-energy ultrasound shock waves (SW), supporting the proof of concept for the development of biomimetic, selective and stimuli-responsive nanodevices for cancer treatment.

## Results and discussion

### EVs characterization

**Fig. 1:**
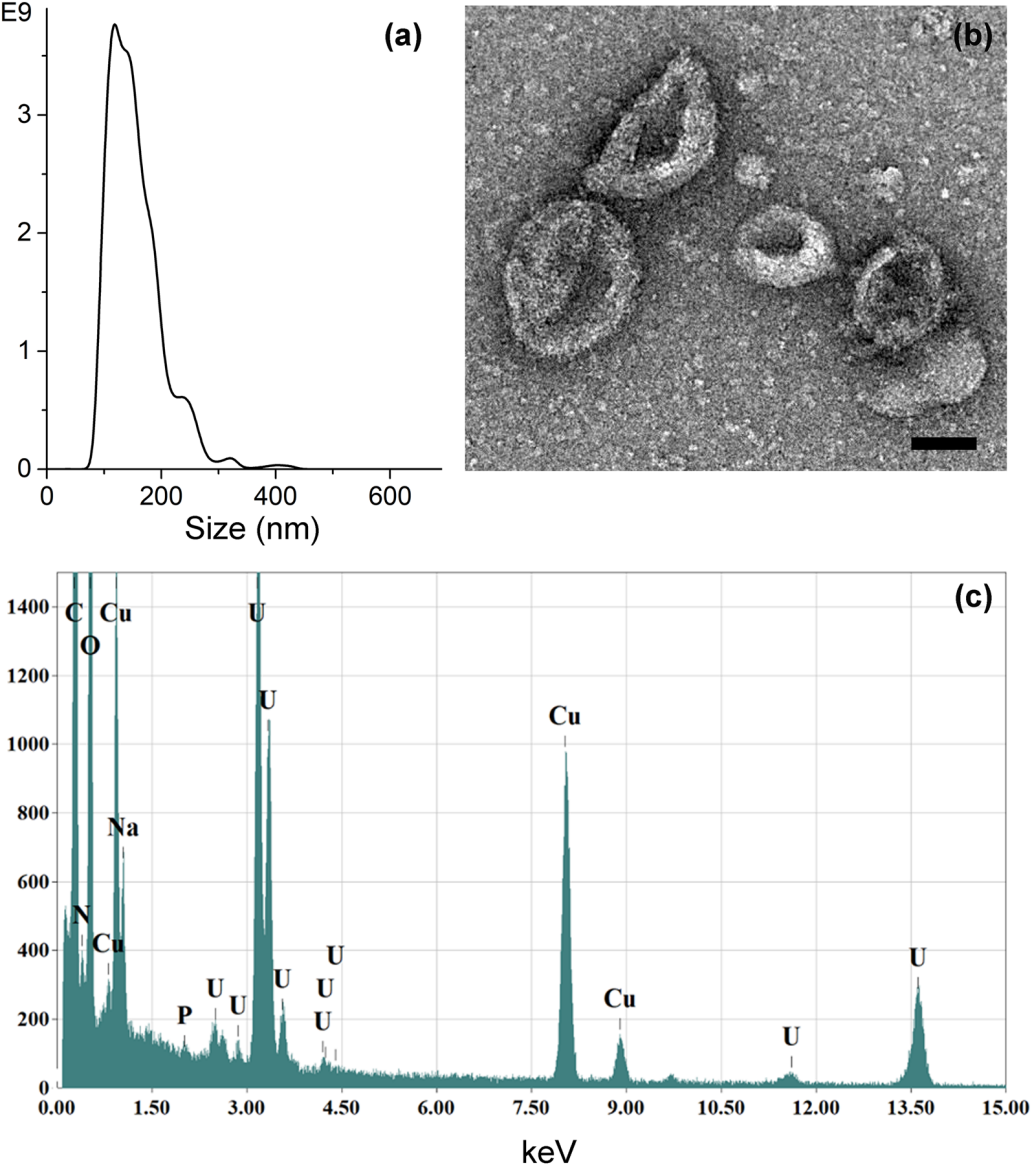
Characterization of lymphocytes-derived EVs. **a** Nanoparticle Tracking Analysis measurement of EVs dispersed in physiological solution. **b** BF-TEM micrograph of EVs negatively stained with uranyl acetate (scale bar: 50 nm) and **c** related EDS spectrum. Results are representative over a broad number (n > 20) of EVs isolation batches

EVs were isolated from lymphocytes’ conditioned supernatants through a sterile differential ultracentrifugation protocol and characterized in terms of size, morphology, composition and concentration. EVs, derived from different isolation rounds, were quantified with both NTA and Bradford assays, obtaining a very uniform population, with a mean particles’ concentration of 1 × 10^11^ ± 1 × 10^10^ particles/mL from NTA analysis and a protein concentration of 160 ± 11 μg/mL from Bradford assays (both concentration values are expressed as mean ± S.E., considering 20 different isolations). NTA measurements also reported the hydrodynamic size distribution of EVs dispersed in physiological solution, identifying a main peak centered at approximately 100 nm, as representatively showed in Fig. 1a. The size and the morphology of EVs were analyzed more in details by TEM technique. TEM image of negatively stained EVs (as representatively depicted in Fig. 1b) showed a population of round-shaped structures with a size distribution in good accordance with NTA measurements. In particular, the presence of vesicles with dimensions of ~ 100 nm or slightly larger and the typical cup-shaped morphology of stained EVs [35] was clearly accounted, while the smallest entities were identified as proteins and lipoproteins co-precipitated during the isolation procedure [36]. The composition of EVs samples was evaluated through Energy Dispersive Spectroscopy. The EDS spectrum reported in Fig. 1c and the EDS elemental maps in Additional file 1: Fig. S1 show the presence of carbon, oxygen, nitrogen and phosphorous, typical elements of lipid vesicles membrane, together with a consistent amount of sodium ascribable to the physiological solution used as dispersant medium.

### ZnO NCs characterization

Thanks to their intrinsic cytotoxic properties widely explored for the treatment of different cancer cell lines [37–40], ZnO crystalline NPs were selected in the present study as the therapeutic payload of the EVs. As extensively reported in the literature [41–43], the cytotoxic potential of ZnO nanostructures is mainly attributed to two main mechanisms, i.e. the production of reactive oxygen species and the NPs dissolution and subsequent release of zinc cations, which are connected and affected by ZnO NPs physico-chemical properties.

**Fig. 2:**
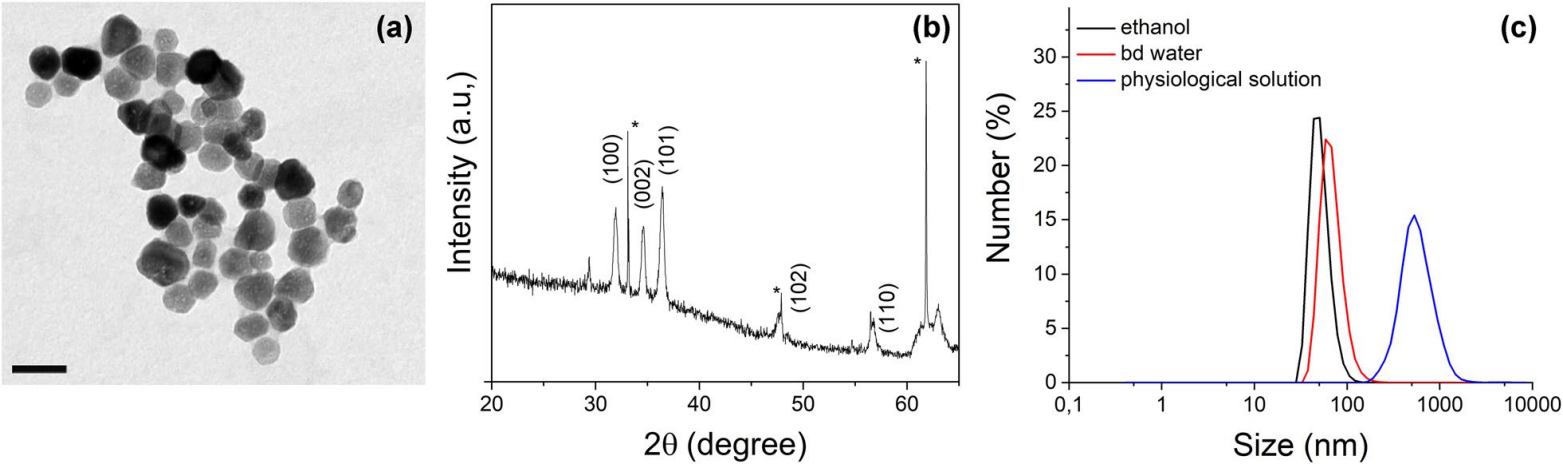
Characterization of amino-functionalized ZnO NCs. **a** Transmission electron microscopy (scale bar: 50 nm). **b** X-ray diffractogram (*: peaks of Si-wafer). **c** Dynamic Light Scattering measurements of ZnO NCs dispersed in ethanol (black line), bd water (red line) and physiological solution (blue line). Results are representative over a broad number (n > 10) of ZnO NCs syntheses and functionalization batches

In the present study, the ZnO NCs were synthesized through a recently-developed solvothermal microwave-assisted method and functionalized with amino groups to further allow the attachment of fluorescent molecules, as previously reported in details [40]. The morphology and dimensions of the obtained ZnO NCs were evaluated through Transmission Electron Microscopy, showing particles with a roughly spherical geometry and an average diameter of about 20 nm (Fig. 2a). XRD measurements (Fig. 2b) confirmed the crystalline nature of the NPs and, specifically, the recorded peaks were indexed as the hexagonal wurtzite phase of ZnO (according to JPCDS card n. 36-1451). The hydrodynamic size distributions of the synthesized ZnO NCs were evaluated through DLS technique, displaying well-dispersed nanocrystals in both ethanol and bd-water with mean diameters of 48 nm and 65 nm, respectively (Fig. 2c). In view of the combination with EVs to form the TNH, the colloidal stability of ZnO NCs in physiological solution was also analyzed, observing a prompt aggregation and the reaching of mean hydrodynamic diameter of 530 nm (Fig. 2c, blue line). This behavior is ascribable to the high ionic strength of the physiological solution, which reduces the repulsive forces between the ZnO nanocrystals, but itis necessary for maintaining the osmolarity of the EVs during TNH assembly. The aggregation of amine-functionalized ZnO NCs in physiological solution is reflected by their Z-potential values, which lowers from + 37 mV in bd water to + 14.5 mV in physiological solution. Despite the reduction, the still positive charge of ZnO NCs could ensure a favorable interaction with the negatively charged membranes of the EVs during the coupling process, as also previously observed [19].

### TNH and TNH^CD20^ assembly and characterization

For the TNH assembly, a convenient coupling ratio between ZnO NCs and EVs was defined on the basis of a simple theoretical model that approximates the TNH components to hard spheres and calculates the theoretical number of nanocrystals that could be geometrically encapsulated within a single EV (Additional file 1: Fig. S2a). Considering a packing density of ~ 74%, corresponding to a cubic close-packing arrangement [44], and the diameters of EVs (100 nm) and of ZnO NCs (20 nm), an amount of about 93 ZnO NCs per EVs was calculated. Based on the actual size of ZnO NCs and EVs experimentally observed by TEM analyses (see Figs. 1b and 2a), it was approximately assumed that each EVs could contain up to 100 nanocrystals and the number ratio was then transformed in a mass ratio, as reported in detail in “Methods” section. The resulting theoretical amount was equal to ~ 1.5 μg of ZnO NCs for each μg of EVs proteins. To favor the probability of collision and thus the interaction between the two components, an excess of ZnO with respect to the theoretical amount was envisaged and a final experimental EVs:ZnO NCs ratio of 1:2 was set.

Concerning the encapsulation procedure, the coupling between ZnO NCs and lymphocytes-derived EVs was obtained through an optimized combination of active and passive loading methods able to maximize the loading efficiency, limit the degradation of EVs membrane and the nanocrystals aggregation. The developed procedure was based on the application of freeze–thaw cycles as active stimulus to destabilize the EVs lipid membrane and favor the coupling with the ZnO NCs [24, 45]. As schematically represented in Additional file 1: Fig. S2b, two freeze–thaw cycles were conducted on a solution containing only EVs, to prevent the mechanical stresses possibly induced by the presence of NCs. Following the addition of ZnO NCs, the sample was then subjected to a quick heating (45 °C for 10 min) to promote a higher fluidity of the already weakened EVs membranes. The procedure was completed with an incubation of 2 h at 37 °C and a further O/N incubation at RT to restore the EVs membranes integrity and microviscosity [13] and complete the encapsulation. In view of in vitro tests, a final centrifugation step to allow the redispersion of the obtained TNHs pellets in the cell culture medium was performed.

The successful coupling of the TNH was primarily evaluated through fluorescence microscopy, quantifying the colocalization between the labeled ZnO NCs and EVs. As shown in Additional file 1: Fig. S3, the two components were imaged in red and green channels, respectively, and then the images in the two different channels were superimposed to analyze the presence of colocalized spots, corresponding to efficiently assembled TNHs nanoconstructs. The obtained average coupling efficiency, expressed as percentage of colocalized spots with respect to the total ZnO signal (%co-ZnO), was equal to 48 ± 6%.

**Fig. 3:**
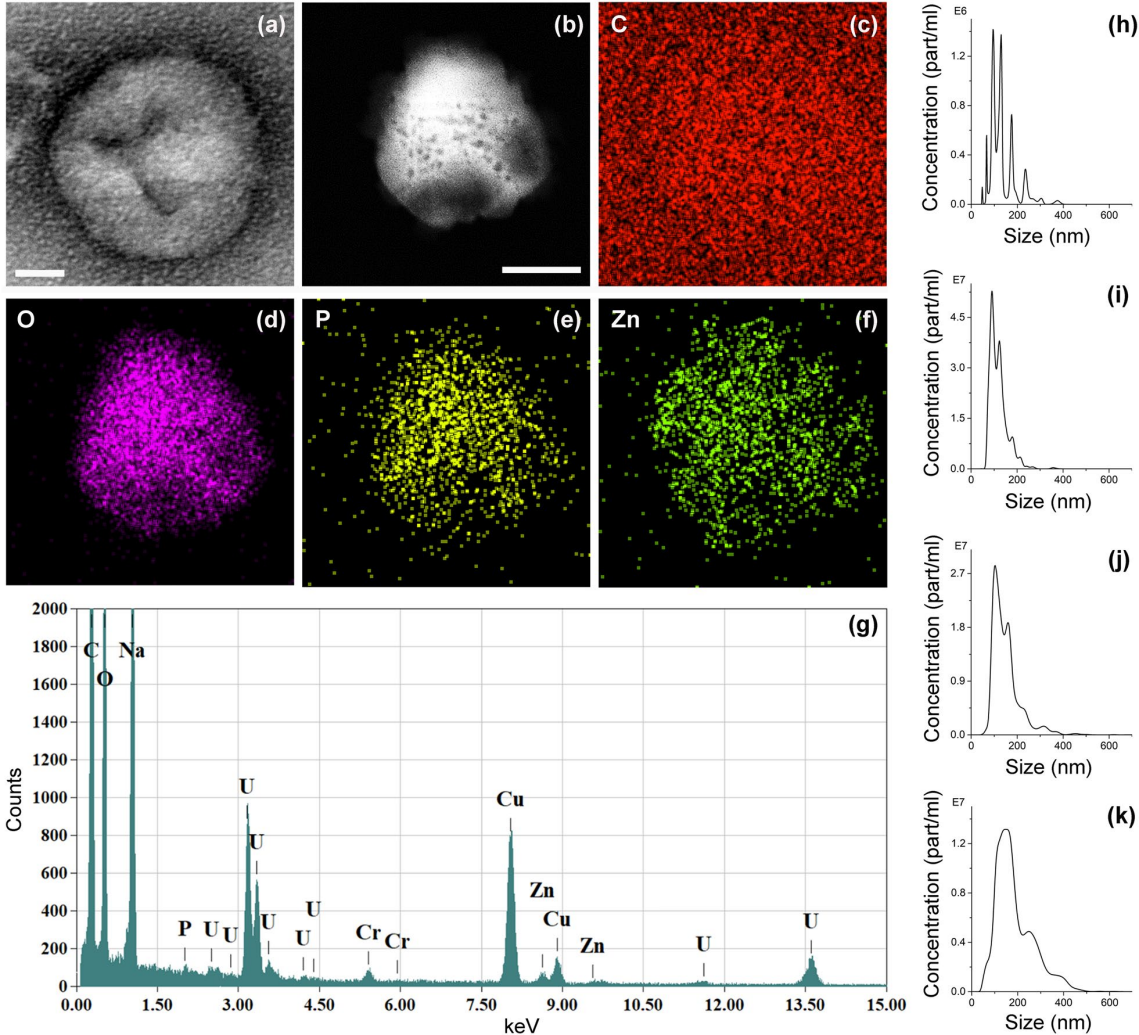
Characterization of TNH nanoconstructs. Representative **a** BF-TEM micrograph of TNHs negatively stained with uranyl acetate (scale bar: 20 nm); **b** ADF-STEM micrograph and **c**–**f** related EDS elemental maps (Scale bars: 200 nm) of Carbon (C), Oxygen (O), Phosphorous (P) and Zinc (Zn) elements; **g** EDS spectrum. Nanoparticle Tracking Analysis measurements of **h** ZnO NCs, **i** EVs, **j** TNHs before and **k** TNHs after the centrifugation step. Samples were analysed in 1:1 (v/v) of 0.1 μm-filtered bd water and physiological solution

The morphology and elemental composition of the TNHs sample were analyzed by electron microscopy. As clearly shown by TEM (see Fig. 3a for a representative image), the original dimension and morphology of EVs were well preserved after their coupling with ZnO NCs. Furthermore, the presence of encapsulated nanocrystals was confirmed by EDS analysis, performed on the area in STEM imaging mode shown in Fig. 3b, together with the related EDS elemental maps (Fig. 3c–f) and spectrum (Fig. 3g). The analyzed object presented a spherical shape and the signals of oxygen, carbon and phosphorous, which are typical elements of lipid membranes, confirming its vesicular nature. In addition, the signal of zinc was detected in the same region, suggesting the encapsulation of ZnO NCs within the EVs.

The colloidal stability of the TNH nanoconstructs was evaluated through NTA technique, measuring the hydrodynamic size distributions of TNHs before and after the final centrifugation step and comparing them with those of EVs and ZnO NCs individual components subjected to the same freeze–thaw cycles and co-incubation steps for consistency. The results, summarized in Fig. 3 from panels h to k, confirmed the tendency of ZnO NCs to aggregate in salt-rich physiological solution, as already highlighted by the DLS measurements (see Fig. 2c). Indeed, the uncoated nanocrystals presented a wide hydrodynamic distribution with multiple peaks centered also at high size values between 200 and 300 nm (Fig. 3h). Conversely, the EVs subjected to the same coupling procedure of the TNH counterpart, displayed good size distribution with a major peak at ~ 100 nm and only minor peaks at higher dimensions (Fig. 3i). This data is quite similar to the one registered for pristine EVs (see Fig. 1a), evidencing that the developed procedure slightly affected the EVs dispersion, causing only a minor extent of aggregation. The TNHs sample before the centrifugation step resulted well-dispersed in the bd water and physiological solution coupling mixture (Fig. 3j), having similar concentration and resembling the distribution of unloaded EVs with only minor peaks at larger diameters (i.e. 150 and 315 nm), thus suggesting the success of the coupling procedure. After the final centrifugation step, a slight broadening of the main peak together with a shift of minor peaks up to 250–300 nm was observed (Fig. 3k). However, despite the partial aggregation probably induced by centrifugation, the TNH maintained the same concentration as before centrifugation and suitable nanometric dimensions for biological applications. It thus constitutes a great improvement with respect to randomly-aggregated ZnO NCs, confirming the stabilization effect provided by the EVs lipid shell.

In the perspective of biological applications, the stability of nanoconstructs in biological medium was also analyzed, monitoring over time the size distribution of TNHs maintained in cell culture medium at 37 °C to mimic the in vitro culture conditions. The NTA measurements, reported in Table 1 in term of mean size and particle concentration, evidenced that the TNHs efficiently maintained their hydrodynamic dimensions up to 48 h, since there were no significant differences comparing the sizes at t_0_ and 1, 24 and 48 h. Even though a contribution ascribable to the detection of smaller medium components must be taken into account, these values confirmed also the EVs stabilizing potential, as their phospholipidic shell efficiently prevent the ZnO NCs aggregation in biological environment, as also widely investigated in a previous study [46]. Moreover, the absence of statistical differences between the concentrations at t_0_, 1, 24 and 48 h suggested a satisfactory robustness of the developed nanoconstruct, with a good over-time retain of ZnO NCs by EVs. This was further confirmed by fluorescence microscopy analysis that indicated a maximum decrease in percentage of colocalized ZnO of about − 20% with respect to the one recorded at t_0_ (Table 1).

**Table 1:** Evaluation of the behaviour of TNHs nanoconstructs in biological media

Stability in cell culture medium					
Time	(a) Mean size (nm)		(b) Concentration (part/mL)		(c) Percentage decrease (as percentage) from initial t_0_ value %co-ZnO
	Medium	TNHs	Medium	TNHs	
t_0_	86 ± 4	128 ± 8	3.88 × 10^9^ ± 3.44 × 10^8^	3.96 × 10^9^ ± 5.57 × 10^8^	–
1 h	79 ± 5	144 ± 4	5.18 × 10^9^ ± 1.05 × 10^8^	6.42 × 10^9^ ± 4.25 × 10^8^	− 17 ± 3%
24 h	78 ± 6	138 ± 3	3.60 × 10^9^ ± 1.50 × 10^8^	7.52 × 10^9^ ± 1.40 × 10^9^	− 20 ± 4%
48 h	83 ± 4	135 ± 13	4.60 × 10^9^ ± 7.73 × 10^8^	5.66 × 10^9^ ± 1.23 × 10^9^	− 15 ± 10%

(d) Clotting time in human recovered plasma				
	Ctrl (plasma)	Plasma + Phys. solution	Plasma + Phys. solution + water	Plasma + Phys. solution + water and TNHs
Time (min)	10.3 ± 0.3	10.4 ± 0.3	10.7 ± 0.5	9.5 ± 0.6

(a) Mean size and (b) concentration of TNHs assessed through Nanoparticles Tracking Analysis at t_0_ and after 1, 24 and 48 h incubation at 37 °C in cell culture medium. (c) Estimation of the coupling efficiency after 1, 24 and 48 h incubation at 37 °C in cell culture medium expressed as percentage decrease of %co-ZnO with respect to t_0_. (d) Plasma clotting time measurements following the incubation with TNHs. The values for plasma alone, plasma/physiological solution, plasma/bd water and physiological solution 1:1 (v/v) are reported as controls. All experiments were conducted at least in duplicate

A first insight of TNHs hemocompatibility was also provided through a simple turbidimetric assay able to track the kinetics of clot formation in human recovered plasma [47]. The results showed no significant differences (p = 0.276) in the clotting time of plasma alone (10.3 ± 0.3 min) or in presence of TNHs (9.5 ± 0.5 min), indicating that treatment with hybrid nanoconstruct did not affect the physiological coagulation process of plasma, hint of good hemocompatibility of TNHs formulation.

After the optimization of ZnO NCs loading procedure, the functionalization of the EVs membrane with anti-CD20 targeting ligands was explored to obtain highly selective TNH^CD20^ nanoconstructs towards CD20+ cells. As schematically represented in Additional file 1: Fig. S2c, the functionalization strategy of TNHs directly involved the use of CD20 antigen as an anchoring site. As a common marker of B cell lines [48], the presence of CD20 antigen on lymphocytes-derived EVs could be reasonably expected and its surface expression on TNH lipid shield was evaluated by flow-cytometry and compared with that of native EVs. The obtained expression values, reported as MFI with respect to the isotype control, were equal to 19 ± 3 and 6.7 ± 1.1 (expressed as mean ± S.E) for native EVs and TNHs, respectively. Although the results confirmed that the expression of CD20 transmembrane protein significantly decrease on lymphocytes-derived EVs during the loading procedure, it was possible to proceed with the functionalization and its effectiveness and selectivity were evaluated through in vitro tests, as described in detail in the next Paragraph. The size distribution of the EVs before and after the functionalization with the anti-CD20 construct where evaluated by NTA (Additional file 1: Fig. S4), showing the maintenance of a monodispersed hydrodynamic size of the samples during the functionalization process and no evident aggregation phenomena.

### Cytotoxicity and targeting capability of TNH versus TNH^CD20^

**Fig. 4:**
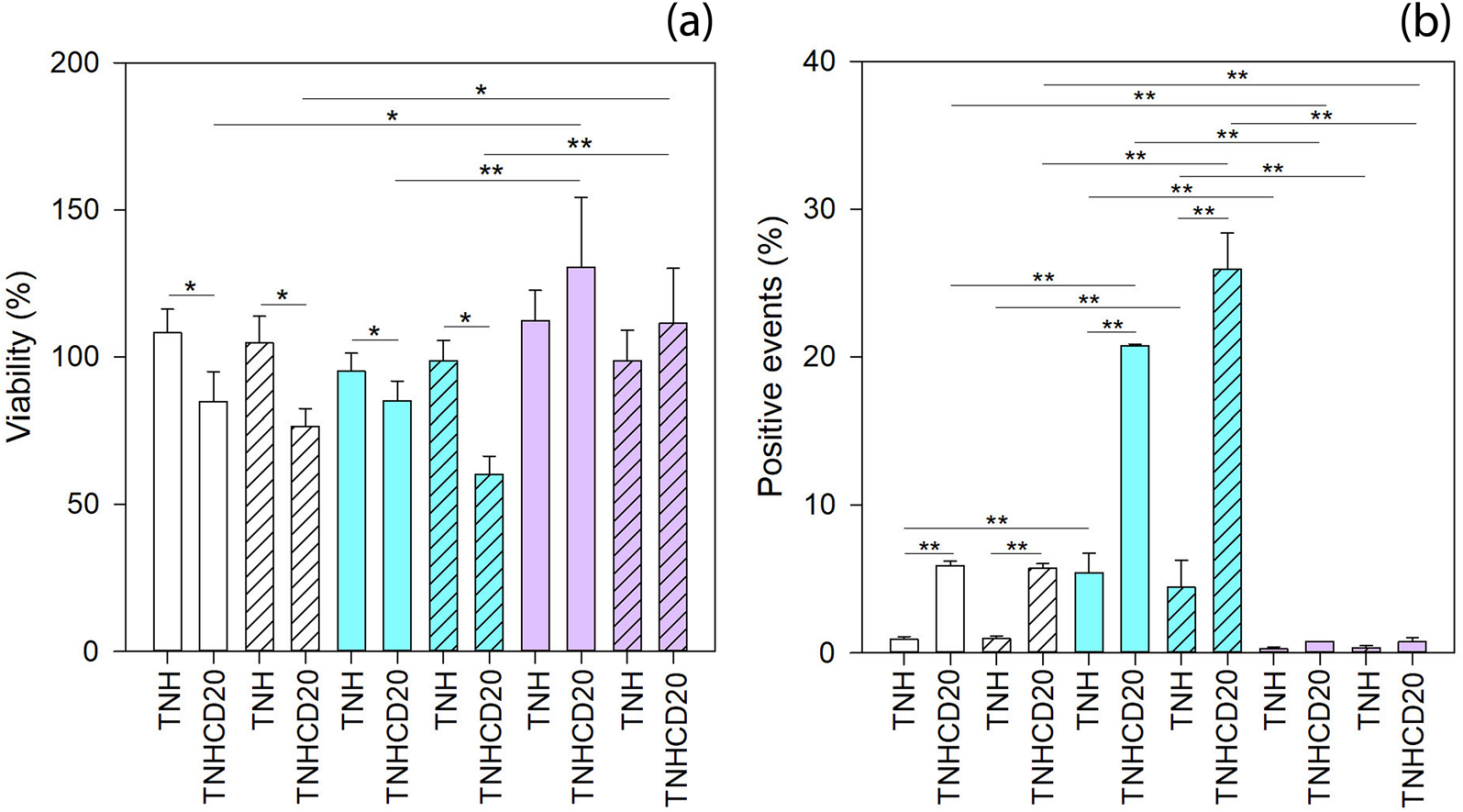
**a** Cytotoxicity and **b** internalization assays of TNH and TNH^CD20^ at 24 (solid colors bars) and 48 (dashed bars) hours in lymphocytes (white bars), Daudi (blue bars) and HL60 (purple bars). Number of assays: n ≥ 4 for cytotoxicity and n ≥ 2 for internalization assay. Statistical analysis: three-way ANOVA. **p ≤ 0.001, *p ≤ 0.05

The results of cytotoxicity assays carried out on the three cell lines, i.e. lymphocytes, Daudi and HL60, at two different time points, 24 and 48 h, are reported in Fig. 4a. Cells viability did not display any statistical differences when considering the time of treatment. On the contrary, significant differences in viability were observed when comparing the different cell lines at the two treatment time points. Moreover, it is worth to note that treating cells with the pristine TNH did not affect cells viability. This result highlights the high biocompatibility of the proposed TNH, similar to what measured using the native lymphocyte-derived EVs at the same concentration and time points on the three cell lines, as previously reported by some of us [25].

Differently, the TNH^CD20^ remarkably decreased the viability of both lymphocytes and Daudi cell lines (p = 0.021 and p = 0.03, respectively), but not that of CD20− HL60 (p = 0.162) if compared to treatment with pristine TNH. Considering the different cell lines treated with TNH^CD20^, it was observed that the CD20− HL60 cells were not affected by the treatment, while the CD20+ cell lines, lymphocytes and Daudi, were slightly impaired (p = 0.004 for HL60 vs. lymphocytes and p < 0.001 for HL60 vs. Daudi).

The results of cytofluorimetric analysis of the cellular interaction with TNH and TNH^CD20^ are reported in Fig. 4b. As already highlighted for the cytotoxicity, there was no statistical difference between the two treatment times of 24 and 48 h. Analyzing the cellular interaction with the nanoconstructs considering the cell lines and the two treatment times, it results that Daudi cells better interacted with the TNH nanoconstruct than lymphocytes and HL60 (p < 0.001). Considering the TNH^CD20^, it interacted more with Daudi than with lymphocytes and HL60 (p < 0.001) and more with lymphocytes than HL60 (p = 0.001). Besides, the most noticeable difference between the pristine and the CD20-targeting nanoconstructs was the striking increase in the interaction of TNH^CD20^ with lymphocytes and Daudi (p < 0.001) compared to that with HL60 cells (p = 0.704), referable to the surface functionalization with antiCD20 antibody. This increase justifies itself in a very intuitive way, since antiCD20-engineered nanoconstruct presents on its surface antiCD20, correctly positioned and exposed, that can efficiently target the CD20 protein expressed on the plasma membrane of both lymphocytes and Daudi cell lines.

At the same time, the TNH^CD20^ did not interact with HL60 cells since these cells do not express CD20 proteins on their plasma membrane [49].

These results clearly demonstrate the following correspondence: the increase of cellular interactions is accompanied by a consequent cytotoxicity of TNH^CD20^ against the target cells, as already described in the comment to Fig. 4a. These results are in agreement with the fact that anti-CD20 monoclonal antibodies, used as the key to the biofunctionalization of the proposed nanoconstruct, actually represent the main treatment to face B cell malignancies triggering cell death even without immune system effector mechanisms [50–55]. Remarkably, it must be emphasized that the treatment with the antiCD20-engineered TNH results not only effective but also selective, as the increment in both interaction and cytotoxicity was more significant considering Daudi cancerous cells instead of lymphocytes.

**Fig. 5:**
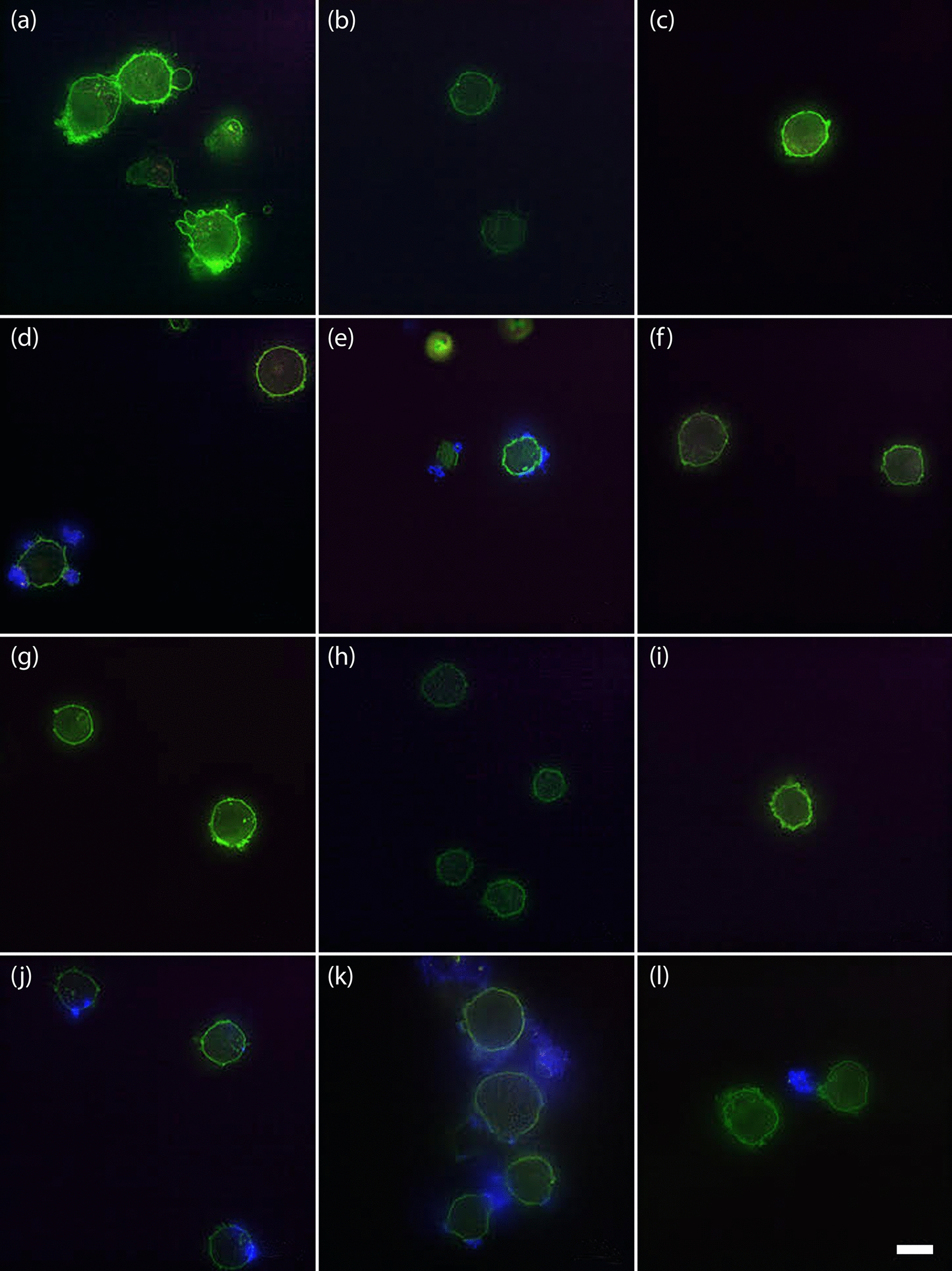
Fluorescence microscopy images of the internalization of the two nanoconstructs. **a** lymphocytes, **b** Daudi and **c** HL60 with TNH at 24 h, **d** lymphocytes, **e** Daudi and **f** HL60 with TNH^CD20^ at 24 h, **g** lymphocytes, **h** Daudi and **i** HL60 with TNH at 48 h, **j** lymphocytes, **k** Daudi and **l** HL60 with TNH^CD20^ at 48 h. Green represents the WGA488-labelled cells’ membranes, blue the secondary antibody of the TNH^CD20^ nanoconstruct, purple the WGA647-labelled EVs and red the Atto550-lebelled ZnO NCs. Scale bar: 10 μm

The fluorescence microscopy analysis (Fig. 5) qualitatively confirmed the interaction of the two nanoconstructs with the tested cell lines for both 24 and 48 h of treatments. The images of the samples treated with the pristine TNH did not show any cellular interactions neither after 24 h (Fig. 5a–c) nor after 48 h (Fig. 5g–i). On the contrary, the interaction of the TNH^CD20^ nanoconstruct with cell membranes of both lymphocytes (Fig. 5d) and Daudi (Fig. 5e) cells was clearly evidenced already after 24 h of treatment by the detection of blue fluorescence, related to the secondary antibody used for antiCD20 functionalization, on the plasma membrane (here in green fluorescence). Referring to the 48 h of treatment, the close interaction was confirmed for lymphocytes (Fig. 5j) and was even more evident for the tumoral Daudi cells (Fig. 5k). In contrast, the fluorescence microscopy on tumoral HL60 cells did not evidenced any interaction of TNH^CD20^ with their plasma membrane (Fig. 5f, l for 24 and 48 h, respectively), as already observed in the case of treatment with the non-functionalized TNH. It is interesting to observe how TNH^CD20^, although present in the treatment well, did not make any contact with the plasma membrane of HL60 cell even after 48 h (Fig. 5l).

### Stimuli-responsive TNH and TNH^CD20^ nanoconstructs with ultrasound shock waves

The obtained results suggest the possibility to employ EVs engineered as TNH and, even better, as TNH^CD20^ as a selective and stimuli-responsive smart nanotool for cancer treatment. Previous results have already confirmed the intrinsic cytotoxic effect of pristine ZnO NCs and the possibility to activate them by ad hoc external stimuli to further enhance their cytotoxic capability [56, 57].

**Fig. 6:**
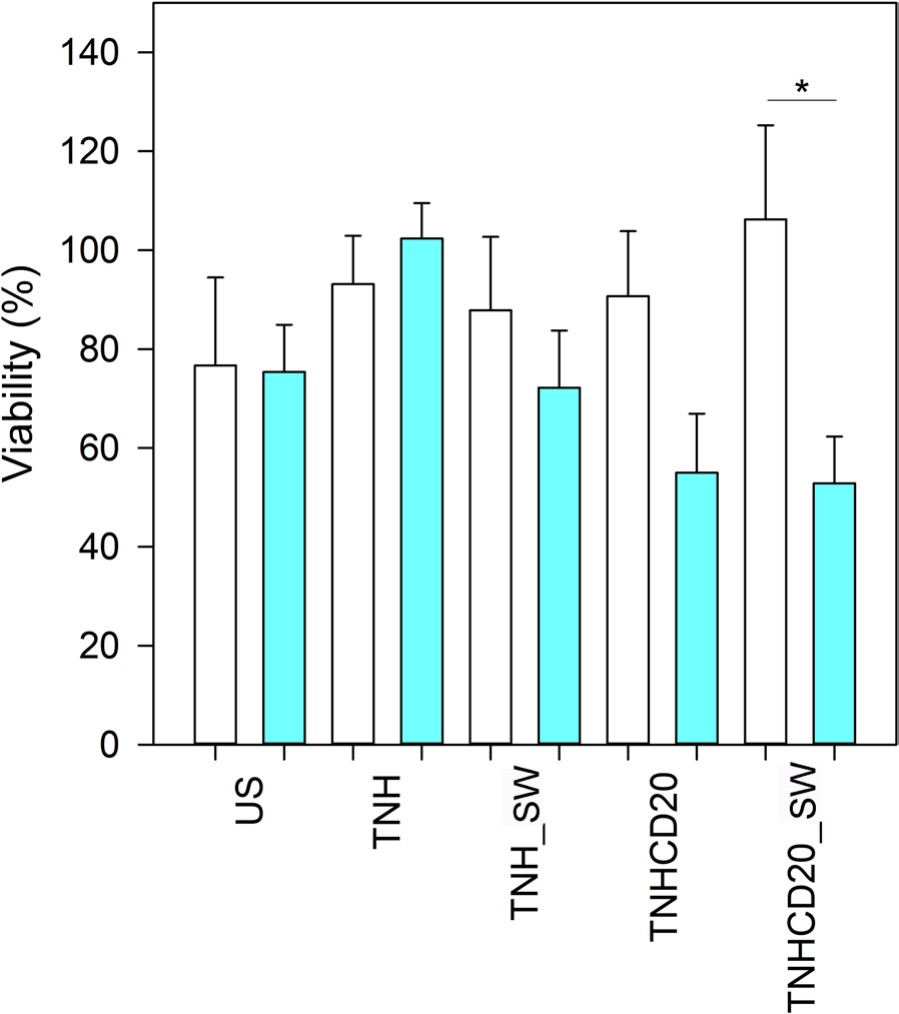
Viability of lymphocytes (white bars) and Daudi (blue bars) 24 h after the SW treatment (i.e. 48 h after the nanoconstruct treatment). Cells were treated with SW alone, TNH, TNH in combination with SW (TNH_SW), TNH^CD20^, and TNH^CD20^ in combination with SW (TNHCD20_SW). Each column is normalized with respect the untreated cells and the experiments were conducted with n = 4. Statistical analysis: three-way ANOVA. **p ≤ 0.001, *p ≤ 0.05. The p value obtained for TNHCD20_SW between B lymphocytes and Daudi cells is p = 0.014

Several physical stimuli, such as light [58, 59], magnetic fields [60, 61] and acoustic waves [56, 62], can be exploited to activate inorganic nanoparticles and induce a cytotoxic effect in living cells. Specifically, periodical acoustic pressure waves, like ultrasounds, or acoustic pulses, like shock waves, can produce acoustic cavitation in water-based media. This consists in cycles of expansion and compression of gas micro-bubbles trapped in the aqueous media in which cells are immersed. Above certain pressure levels, the implosion of these gas bubbles is produced (inertial cavitation) causing the release of a high amount of energy in the dispersing medium. Inertial cavitation locally causes very high increases of temperature and pressure, which in turns can induce the formation of water-derived radicals and mechanical damages to cellular compartments and membrane [62]. Indeed, the possibilities of acoustic stimulation are many as well as challenging to be optimized. Continuous or pulsed ultrasound irradiations, highly focused or unfocused transducers use, high or low-intensity ultrasound have been broadly exploited in the literature [62]. Of prominence importance is however the use of nanomaterials, the presence of which can on the one hand reduce the cavitation threshold, thus allowing to employ low intensity ultrasound or shorter application times [63, 64]. On the other hand, nanomaterials can be opportunely functionalized and engineered to allow specific targeting to selected cells or tissues, thus favouring the cytotoxic action of such nanoparticles-assisted ultrasound in a specific region [65]. Motivated by the above-mentioned rationale, as a proof of concept, we proved the cytotoxic effect of TNH and TNH^CD20^ when activated with high-energy ultrasound shock waves (SW) on in vitro healthy and cancer cell line, i.e. lymphocytes and Daudi cells. As shown in Fig. 6, 48 h after the treatment, the SW alone caused a slight decrease of the cell proliferation in both lymphocytes and Daudi cells. This result accounts for the good choice of the irradiation parameters (intensity of 12.5 MPa, number of used shots—250, number of SW applications, 3 times/day, one treatment every 3 h), in terms of stimuli safety. Noticeably, the treatments with TNH^CD20^ presented a higher, even though not significant, cytotoxic effect on Daudi cancer cell line with respect to that on the healthy counterpart (lymphocytes). Strikingly, a larger and statistically significant difference (p = 0.014) in cell viability between Daudi and lymphocytes was measured treating cells with the combination of both TNH^CD20^ and SW.

Despite the full understanding of the biomolecular mechanism of the induced cell death is still the focus of our further studies and characterizations, we recall here that the observed enhanced cytotoxicity is due to the concomitant administration of the SW and of the targeting TNH^CD20^. In particular, the observed cytotoxicity could be caused by the combination of various concurrent effects leading to the mechanical injury of the cell structure [56]: (i) an enhanced acoustic bubble cavitation and (ii) the “nanoscalpel effect” supported by the preferred interaction of the TNH^CD20^ construct with the Daudi cell line. Finally the viability of the treated cells may also have been affected by an electric charge imbalance, due to the piezoelectric behavior of ZnO nanostructures [66]. The present study suggests the applicability of our hybrid nanoconstruct as a targeted anticancer tool, which not only demonstrated to be more cytotoxic to Burkitt’s lymphoma cells, but further highlighted its potential for an externally and on-demand activated therapy.

## Conclusions

The engineering of extracellular vesicles is a hot topic in the current research. The driving idea is, from the one hand, to take chance of their natural cargo shuttling capabilities and specific homing to certain tissues. On the other hand, a nanotechnological engineering allows to expand enormously their diagnostic capabilities and therapeutic activity against diseased cells, like cancer ones, or even improve their selective targeting specificity.

Here we demonstrated the possibility to efficiently engineer the EVs derived from healthy cells using inorganic nanoparticles and monoclonal antibodies. In particular, we propose an efficient active method based on freeze–thaw cycles and mild heating to load B-cell derived EVs with a nanotherapeutic stimuli-responsive cargo, i.e. ZnO NCs. The resulting re-engineered EVs are thus called Trojan Nano Horses (TNHs), to convey the concept of biomimetism provided by EVs and the cytotoxic potential given by the ZnO NCs on this novel nanoconstructs. Such TNHs show good hemocompatibility and high colloidal stability up to 48 h in cell culture medium. Afterwards, TNHs are modified at their surface with anti-CD20 monoclonal antibodies to obtain a selective targeting against lymphoid cancer cell line, i.e. Daudi cells. The in vitro characterization has shown the high TNH biocompatibility and the remarkable selectivity of anti-CD20 engineered nanoconstructs (TNH^CD20^) towards the target CD20+ lymphoid Daudi cell line compared to the CD20- cancerous myeloid cells (HL60) and the healthy counterpart (lymphocytes). Furthermore, an enhanced cytotoxicity of TNH^CD20^ directed against Daudi cancer cells was demonstrated after the nanoconstructs activation with high-energy ultrasound shock waves. The obtained hybrid nanoconstructs can be thus on-demand activated by an external stimulation, here acoustic waves, to efficiently exploit a cytotoxic effect conveyed by the ZnO NCs cargo against selected cancer cells, while remaining highly biocompatible towards healthy B cells.

## Methods

### Cell cultures

The three cell lines used were all cultured in conformity with the sterile technique and the standard mammalian cell culture protocols under a 5% CO_2_ atmosphere at 37 °C.

Lymphocyte cell line (IST-EBV-TW6B) was purchased from the cell bank IRCCS AOU San Martino IST (Italy). Cells were cultured in advanced RPMI 1640 culture medium (Gibco) with 20% of heat inactivated fetal bovine serum (FBS, Gibco), 1% penicillin/streptomycin (P/S, Sigma) and 1% of l-glutamine 200 mM (Lonza) in 75 cm^2^ not treated cell culture flasks (Corning) maintaining the cell density between 9 × 10^4–5^ cells/mL.

Daudi cells (ATCC® CCL­213™), originating from a Burkitt’s lymphoma patient, were obtained from American Type Culture Collection (ATCC). Cells were grown in RPMI 1640 culture medium (ATCC) supplemented with 10% of heat inactivated FBS (ATCC), 1% P/S (Sigma) in 75 cm^2^ not treated cell culture flasks (Corning) with a cell density between 3 × 10^5–6^ cells/mL.

HL60 cells (ATCC® CCL-240™), from an acute myeloid leukemia patient, were purchased from ATCC. They were maintained in Iscove’s Modified Dulbecco’s Medium (Sigma) with 20% heat inactivated FBS (Sigma), 1% Glutamine (Sigma), 1% P/S (Sigma) in 75 cm^2^ not treated cell culture flasks (Corning), adjusting cell density to 1 × 10^5–6^ cells/mL.

### EVs isolation and characterization

EVs were isolated from the conditioned media of the lymphocytes cell line grown in RPMI supplemented with 20% EVs-depleted FBS, 1% glutamine and 1% P/S after 72 h of culture. The depleted FBS was the supernatant collected from the overnight ultracentrifugation at 100,000×*g* (Optima Max-XP Ultracentrifuge with MLA-50 rotor, Beckman Coulter) at 4 °C of FBS.

EVs were produced by plating 1.5 × 10^5^ lymphocytes/mL in a total volume of 200 mL of medium complemented with depleted FBS in 75 cm^2^ untreated flasks and left in culture for 3 days at 37 °C under a 5% CO_2_ atmosphere. Just before the EVs extraction, lymphocytes viability was assessed via Trypan-blue (VWR) method using a TC20 TM automated cell counter (BiO-Rad Laboratories), and only samples with viability over 90% were processed to reduce the probability of apoptotic bodies’ recovery. The EVs isolation protocol is adapted from the sterile differential ultracentrifugation protocol described by Thery et al. [67]. Cell culture medium was collected in 50 mL tubes and centrifuged 10 min at 150×*g* at 4 °C to remove cells. Supernatants were collected and centrifuged 20 min at 2000×*g* at 4 °C to remove dead cells and cell debris. The supernatants collected were centrifuged again for 30 min at 10,000×*g* at 4 °C to discard aggregates of biopolymers, apoptotic bodies, and other structures with higher density than EVs. Supernatants were collected again, placed in ultracentrifuge polypropylene tubes (32 mL Optiseal tubes, Beckman Coulter) and ultracentrifuged at 100,000×*g* for 70 min at 4 °C. The obtained pellet was resuspended in sterile, cold, 0.1 μm filtered phosphate buffered saline (PBS) solution and ultracentrifuged for further 60 min at 100,000×*g* at 4 °C. The pellet, which contained EVs, was resuspended in 600 μL of sterile, cold, 0.1 μm filtered physiological solution (0.9% NaCl, NovaSelect), aliquoted in 50 μL cryovials and stored at − 80 °C for further uses.

The concentration and the size distribution of collected EVs were measured by nanoparticle tracking analysis (NTA) technique with a NanoSight NS300 (Malvern Panalytical) equipped with λ = 505 nm laser beam and a NanoSight syringe pump. Samples were diluted in a final volume of 500 μL of 0.1 μm-filtered physiological solution to meet the ideal particles per frame value (20–100 particles/frame). Different EVs aliquot were measured by capturing three videos of 60 s with an infusion rate of 50 a.u, and a camera level value between 14 and 16. The collected videos were then analyzed by the NTA 3.4 software (Malvern Panalytical), setting the detection threshold at 5.

The protein content of isolated EVs was measured by Bradford assay as described in literature [67]. Bradford reagent (Bio-Rad) was diluted 1:5 in bd water and added to EVs samples, diluted 1:2 in 0.1 μm-filtered PBS, and serially diluted bovine serum albumin (BSA, Sigma Aldrich) standards with known concentrations. The absorbance at 590 nm was then recorded using a microplate spectrophotometer (Multiskan GO, Thermo Fisher Scientific) and the protein concentration of EVs samples was extrapolated comparing their absorbance values with the calibration curve made on BSA standards. All samples were analyzed in triplicate.

The EVs morphology was analyzed trough Transmission Electron Microscopy (TEM) using a JEOL JEM-1400Plus TEM, with thermionic source (LaB6), operated at 120 kV. For TEM analyses, a drop of the sample solution was placed on a copper grid, 150 mesh, coated with amorphous carbon film; then, to highlight the EVs morphology, the EVs were stained before observation with a solution of 1% uranyl acetate in water. Energy Dispersive X-ray Spectroscopy was performed with the same instrument equipped with a JEOL-JED-2300 Energy Dispersive Spectroscopy (EDS) silicon drift type detector (area 30 mm^2^).

To evaluate the presence of the CD20 surface antigen on EVs’ membranes, vesicles were adsorbed on Aldehyde/Sulfate Latex Beads, 4% w/v, 3 µm (Thermo Fisher) and analyzed by flow cytometry using the Guava Easycyte 6-2L flow cytometer (Merck Millipore). In details, 10 μL of latex beads were coupled for 15 min at RT with a sample of EVs containing 5 μg of protein. Then, PBS was added to a final volume of 1 mL and the coupling continued for 2 h at RT on a tube rotator with fixed speed of 20 min^−1^. To saturate any free binding site of the beads, 110 μL of PBS/1 M glycine were added and incubated for 30 min at RT. Then, samples were centrifuged for 3 min at 4000 rpm, the supernatants were discarded, and the bead pellets were resuspended in 1 mL PBS/0.5% BSA. Beads were washed three times before the incubation with CD20-PE antibody (Miltenyi Biotec) and the respective isotype control. Unstained beads were used to adjust instrument voltages and gate bead population to exclude debris and impurity derived from buffer solution. 5 × 10^3^ gated events were acquired in very low modality (0.12 μL/s flow rate) and the PE signal was excited with blue laser (488 nm). Results were analyzed with Incyte Software in term of median fluorescence intensity (MFI) of the antigen minus the MFI of the isotype control [68, 69]. Each experiment was repeated three times (n = 3).

### ZnO NCs synthesis, functionalization and characterization

ZnO NCs were synthesized through a microwave-assisted solvothermal approach [40]. In details, the solution of zinc precursor, i.e., zinc acetate dihydrate (99.99% Sigma Aldrich) 0.1 M in methanol, was stirred directly in the microwave-reactor vessel. 0.48 mL of bidistilled (bd) water were added to initiate the nucleation and then a KOH solution (≥ 85% pellets, Sigma-Aldrich, 0.2 M in methanol) was rapidly added. The resulting solution, with an overall pH of 8, was put in the microwave oven (Milestone START-Synth, Milestone Inc) at 60 °C for 30 min, under temperature and pressure control and with a maximum microwave power of 150 W. Upon completion of the reaction, the obtained colloidal suspension was cooled down to room temperature (RT) and centrifuged (3500×*g* for 10 min) to remove the unreacted compounds. The as-obtained pellet was dispersed in fresh ethanol through sonication and the washing step was repeated two more times.

The ZnO NCs were further functionalized with amino groups (–NH_2_) [40]. Briefly, the synthesized ZnO NCs dispersed in ethanol were heated to 70 °C in a round glass flask under continuous stirring and nitrogen gas flow. After approximately 15 min, the functionalizing agent (3-amminopropyltrimethoxysilane, APTMS, 97% Sigma Aldrich), was added in a molar ratio of 10 mol% with respect to the total ZnO amount. The reaction was carried out in reflux condition under nitrogen atmosphere for 6 h and then washed twice, in to order to remove unbound APTMS molecules, by centrifuging (10,000×*g* for 5 min).

The morphology of ZnO NCs was evaluated through Transmission Electron Microscopy using a Jeol JEM-1011 transmission electron microscope operated at 100 kV of acceleration voltage. The crystalline structure of ZnO NCs was investigated by X-ray diffraction (XRD) measurements using a Panalytical X’Pert diffractometer (Malvern Panalytical) in configuration θ–2θ Bragg–Brentano equipped with a Cu-Kα radiation source operating at 40 kV and 30 mA. The sample in ethanol was deposited drop by drop on a silicon wafer and analyzed collecting the spectrum in the range of 20°–65° with a step size of 0.02° and an acquisition time per step of 100 s. The hydrodynamic size and the z-potential of the ZnO NCs were determined using the dynamic light scattering (DLS) technique with a Zetasizer Nano ZS90 (Malvern Instruments). The measurements were performed at RT on samples with concentration of 100 μg/mL and sonicated for 10 min before the acquisition.

### TNH assembly and characterization

The EVs:ZnO NCs ratio of 1:2 used during TNH assembly was calculated starting from a model which estimates the maximum number of nanocrystals that could be geometrically encapsulated within a single vesicle, as schematically represented in Additional file 1: Fig. S2a. Considering the EVs concentration as part/mL and μg/mL obtained from NTA and Bradford techniques respectively, the maximum theoretical number of ZnO NCs (indicated as n°_ZnO NCs_), corresponding to a fixed amount of μg of EVs, was calculated as follows:

$$({\text{n}}^\circ_{{{\text{ZnO}}\;{\text{NCs}}}} )_{{\upmu {\text{g}}\;{\text{EVs}}}} = ({\text{n}}^\circ_{{{\text{ZnO}}\;{\text{NCs}}}} )_{{{\text{EV}}}} \cdot {\text{n}}^\circ_{{{\text{EVs}}\;{\text{per}}\;\upmu {\text{g}}}} \cdot\upmu {\text{g}}\;{\text{EVs}}\quad {\text{where}}\quad {\text{n}}^\circ_{{{\text{EVs}}\;{\text{per}}\;\upmu {\text{g}}}} = \frac{{{\text{Conc}}_{{{\text{EVs}}@{\text{NTA}} \left[ {{\text{part}}/{\text{mL}}} \right]}} }}{{{\text{Conc}}_{{{\text{EVs}}@{\text{Bradford}} \left[ {\upmu {\text{g}}/{\text{mL}}} \right]}} }}.$$

Finally, the mass of a single particle was calculated considering its volume (ie the volume of a sphere with diameter equal to the ZnO NC diameter, d_ZnO_) and the ZnO density (ρ_ZnO_ = 5.606 g/cm^3^) and the obtained value was used to determine the corresponding NCs amount expressed as μg:

$$\upmu {\text{g}}\;{\text{ZnO}}\;{\text{NCs}} = ({\text{n}}^\circ_{{{\text{ZnO}}\;{\text{NCs}}}} )_{{\upmu {\text{gEVs}}}} \cdot{\text{mass}}_{{{\text{ZnO}}\;{\text{NC}}}} \quad {\text{where}}\quad {\text{mass}}_{{{\text{ZnO}}\;{\text{NC}}}} = \left( {\frac{\uppi }{6}{\text{d}}_{{{\text{ZnO }}\;{\text{NC}}}}^{3} \cdot10^{ - 21} } \right)\cdot\rho_{{{\text{ZnO}}}} \cdot10^{6} .$$

The model was then amended on the basis of experimental observations, as discussed in detail in “Results and discussion” section, and finally an excess of 10 μg of amino-functionalized ZnO NCs were combined with an amount of EVs corresponding to 5 μg of protein measured by Bradford assay. The encapsulation process was performed in a 1:1 (v/v) solution of 0.1 μm-filtered bd water and physiological solution, with a final concentration of 80 μg/mL for ZnO NCs and 40 μg/mL for EVs. As schematically represented in Additional file 1: Fig. S2b, opportunely labeled EVs dispersed in physiological solution were rapidly frozen in liquid nitrogen for 3 min and thawed at RT for 15 min. The freeze–thaw cycle was repeated twice and then the corresponding amount of ZnO NCs in bd water was added. The obtained mixture was incubated under continuous agitation (250 rpm) at 45 °C for 10 min, at 37 °C for 2 h and then overnight (O/N) at RT. In order to redisperse the obtained TNHs in media suitable for in vitro tests, a final step of centrifugation was performed. The samples were centrifuged at 5000×*g* for 5 min, suspended in the cell culture medium and redispersed by vortexing for 3 min.

The coupling efficiency was evaluated through fluorescence microscopy. The amino-functionalized ZnO NCs were labeled with Atto 550-NHS ester (λ_Ex_ = 554 nm, ATTO-Tech), by adding 4 μg dye each mg of ZnO NCs suspension in ethanol; the solution was stirred in dark O/N and then washed twice. EVs, diluted 1:2 in physiological solution were labeled with Wheat Germ Agglutinin (WGA) conjugated with Alexa Fluor 488 (WGA488, λ_Ex_ = 495 nm, Thermo Fisher) by adding 1 μL of dye (100 μg/mL in PBS) for each EVs aliquot containing approximately 1 × 10^10^ particles. The solution was kept under agitation (180 rpm) in dark at 37 °C for 30 min and then purified from unbound dye molecules with 50 kDa Amicon Ultra 0.5 centrifugal filter (Merck Millipore). The samples were analyzed using a wide-field fluorescence-inverted microscope (Eclipse Ti-E, Nikon) equipped with a super bright wide-spectrum source (Shutter Lambda XL), a high-resolution camera (Zyla 4.2 Plus, 4098 × 3264 pixels, Andor Technology) and an immersion oil 100× objective (Nikon). The collected images were analyzed with the colocalization tool of NIS-Element software (NIS-Element AR 4.5, Nikon). In brief, the spots in red and green channels (corresponding to ZnO NCs and EVs respectively) were counted and then a merge of the two images was performed, counting the spots in which the two fluorescence signals resulted superimposed. The percentage of colocalization with respect to the ZnO NCs (%co-ZnO) was then calculated doing the ratio between the number of colocalized spots and the total number of red spots. The analysis was performed on 9 regions of interest (ROIs) to evaluate the mean %co-ZnO and the results of 5 different samples were averaged to obtain the coupling efficiency at the end of TNHs assembly process. The same colocalization procedure was used to evaluate the maintenance of coupling efficiency after incubation in cell culture medium samples (advanced RPMI + 20% EVs-depleted FBS, Gibco). Four different samples were analyzed at t_0_ and after 1, 24 or 48 h of incubation and the results were expressed as percentage decrease of %co-ZnO with respect to t_0_.

To evaluate the TNHs morphology, a drop of the sample solution was placed on a copper grid, 150 mesh, coated with amorphous carbon film and the sample was stained with a solution of 1% uranyl acetate in water for 30 s. TEM analysis in Bright Field mode were performed using a JEOL 1011 operated at 100 kV. Annular Dark-Field (ADF) imaging in Scanning Transmission Electron Microscopy (STEM) mode and EDS analysis were performed using a TEM JEM-1400 Plus, with thermionic source, operated at 120 kV of accelerating voltage and equipped with a JEOL-JED-2300 EDS silicon drift type detector (detector area 30 mm^2^).

The size distribution of TNHs in bd water and physiological solution 1:1 (v/v) was assessed by NanoSight NS300 equipped with NanoSight syringe pump. The samples were diluted 1:5 and three videos of 60 s were recorder with camera level and detection threshold of 16 and 5, respectively. The size distribution of TNHs in advanced RPMI (Gibco) supplemented with 20% EVs-depleted FBS (Gibco) was measured in static conditions using the O-ring top plate cell with manual syringe connection. TNHs resuspended in cell culture medium (ZnO concentration 100 µg/mL) and medium alone as reference were diluted 1:10 in bd water and physiological solution 1:1 (v/v) and three videos of 30 s were acquired, advancing manually the samples between them. The camera level and detection threshold values were set at 15 and 6, respectively. At least two independent experiments were performed.

TNHs hemocompatibility was preliminarily evaluated through a simple turbidimetric assay as previously reported [47], using Na-citrate human recovered plasma (Zen Bio) and calcium chloride (CaCl_2_ 0.025 M from HYPHEN BioMed) as clotting agent. Briefly, 75 µL of plasma for each sample were aliquoted in a 96 well plate and mixed with 75 µL of TNHs samples at concentration 75 µg/mL in bd water and physiological solution 1:1 (v/v). To monitor the dispersant influence, controls with the addition of 75 µL of physiological solution or bd water and physiological solution 1:1 (v/v) were also performed. Coagulation was started adding 75 µL of CaCl_2_, and the absorbance at 405 nm was measured through a microplate UV–VIS spectrophotometer every 30 s for 45 min at constant T = 37 °C. Three replicates per sample were averaged to obtain the mean absorbance at each time point and the coagulation time, was calculated as the time corresponding to the half maximal absorbance (t_1/2_). Four independent experiments were conducted and the results were expressed as mean ± S.E.

CD20 expression on TNH surface was evaluated by flow cytometry as described in “EVs isolation and characterization” section. Each experiment was repeated three times (n = 3).

### TNH functionalization with targeting antibodies

To obtain TNH^CD20^ samples, vesicle membranes were functionalized with anti-CD20 antibody as previously reported in [25] and schematically represented in Additional file 1: Fig. S2c. After the O/N co-incubation step, TNH samples corresponding to 5 μg of EVs proteins were mixed with functionalizing antibodies, added in three consecutive incubation steps (1 h at RT on a tube rotator with fixed speed of 20 min^−1^ each). In details, half of the EVs protein content measured by Bradford assay was considered equal to CD20 antigen and anti-CD20 antibody (Rituximab, Cat. n° TAB-016, Creative Biolabs, 5 mg/mL in PBS) was added in a molar ratio 4:1 with respect to the assumed antigen concentration, working in a large excess to favor antibody-antigen interaction. Then, anti-human secondary antibody (AffiniPure F(ab′)_2_ Fragment Goat Anti-Human IgG, Fcγ fragment specific, Jackson ImmunoResearch or AMCA AffiniPure F(ab′)_2_ Fragment Goat Anti-Human IgG, Fcγ fragment specific, Jackson ImmunoResearch, λ_Ex_ = 450 nm) was added as cross-linker in molar ratio secondary Ab: anti-CD20 = 1:1. Finally, a second aliquot of anti-CD20 antibody was added in the same amount as the first incubation step. After the third hour of incubation, TNH^CD20^ samples were collected and centrifuged (5000×*g* for 5 min) to remove unbound antibodies and resuspended in cell culture medium suitable for in vitro tests.

### Cytotoxicity assay of TNH and TNH^CD20^

To evaluate the viability of lymphocytes, Daudi and HL60 cell lines treated with 5 μg/mL of TNH and TNH^CD20^ (considering the EVs protein content), after the centrifugation step at 5000×*g*, the two TNH samples were resuspended in the required volume of cell culture medium. Then, 2 × 10^5^ cells for each mL of treatment were centrifuged at 130×*g* for 5 min for Daudi and HL60 and at 150×*g* for 5 min for lymphocytes, and the supernatants replaced with the treatment solutions of TNH and TNH^CD20^. A total volume of 100 μL was plated for each well in a 96-well flat-bottom plastic culture plate (Greiner Bio-one, 96 Well for suspension culture). After 20 and 44 h of incubation 10 μL of WST-1 reagent (CELLPRO-RO Roche) was added to each well and, after further 4 h of incubation, the formazan absorbance was detected at 450 nm by the microplate spectrophotometer using a 620-nm reference. All the experiments were carried out at least four times for each cell line and results were normalized to the untreated control.

### Cytofluorimetric analysis of TNH and TNH^CD20^ internalization

For the uptake evaluation of TNH and TNH^CD20^, the amino-functionalized ZnO NCs were labeled with Atto 647-NHS ester (λ_Ex_ = 647 nm, ATTO-Tech) fluorescent probe as previously described, and the preparation of the TNH performed as described above. After the centrifugation steps, the two TNHs were resuspended in cell medium. 2 × 10^5^ lymphocytes, Daudi and HL60 cells for each mL of treatment were centrifuged and the pellets were resuspended in the TNHs’ solutions. The experiment was carried out five times for TNH and in duplicate for TNH^CD20^. Data from untreated cells were used as reference.

Cells were cultured into not treated 96 well plates, 250 μl for each well. After 24 and 48 h of incubation, the contents of the different wells were collected and washed twice in PBS and resuspended in 350 μL of PBS for the 24 h and 500 μL for the 48 h cytofluorimetric analysis. 1 × 10^4^ events were acquired with the flow cytometer with 0.59 μL/s flow rate, excluding cell debris. The analyses were performed using the red laser (λ_Ex_ = 642 nm). Positive events were characterized by a shift of Red-R fluorescence intensity (emission filter 661/15) and the percentages of positive events were evaluated with respect to untreated cells using Guava InCyte Software (Merck Millipore).

### Fluorescence microscopy imaging of TNH and TNH^CD20^ internalization

For the fluorescence microscopy analysis, EVs were labelled with Wheat Germ Agglutinin (WGA) conjugated with Alexa Fluor 647 (WGA647, λ_Ex_ = 650 nm, Thermo Fisher), ZnO NCs with Atto 550-NHS ester (λ_Ex_ = 554 nm, ATTO-Tech), and the TNH^CD20^ nanoconstruct was assembled using the AMCA AffiniPure F(ab′)_2_ Fragment Goat Anti-Human IgG, Fcγ fragment specific as secondary antibody.

Samples were treated with the same protocol used for the cytofluorimetric analysis and plated in a volume of 100 μL. After 24 and 48 h of culturing at 37 °C, 5% CO_2_ in 96 well plates, the content of each well was collected, centrifuged, resuspended in 40 μL of the correspondent medium. The 40 μL drop was spotted in a 8-well chamber slide (Thermo Scientific™ Nunc™ Lab-Tek™ II CC2™ Chamber Slide System) and placed at 37 °C, 5% CO_2_ for 30 min to allow the attachment of the cells. After that, cells were fixed using 250 μL of Image-iT™ Fixative Solution (4% formaldehyde, methanol-free, Thermo Scientific) for 10 min, washed in PBS and cells’ membranes were labelled by incubating cells with 1.25 μL of WGA conjugated with Alexa Fluor 488 (WGA488, λ_ex_ = 495 nm, Thermo Fisher) for 10 min and washed two other times in PBS. Images were acquired using a wide-field fluorescence-inverted microscope using an immersion oil 100× objective.

### Shock waves treatment

The cytotoxic effects of shock waves combined with both TNH and TNH^CD20^ treatments were evaluated on Daudi and lymphocytes cell lines.

After the centrifugation steps, the TNH and TNH^CD20^ were resuspended in cell culture medium. 2 × 10^5^ lymphocytes and Daudi for each mL of treatment were centrifuged and the pellets were resuspended in the TNH and in the TNH^CD20^ solutions and seeded into 96 well plates, 100 µL for each well. Untreated cells were used as reference. After 24 h of incubation cells were treated with multiple SW (3 times/day, one treatment every 3 h). Each SW treatment was composed by 250 shots of 12.5 MPa, 4 shot/s, SW were generated by PW^2^ device from Richard Wolf. The cell viability was measured 24 h after the SW treatment with the WST-1 assay.

### Statistical analysis

Plotted data are mean ± S.E. The statistical analysis between the treatment groups was performed by using the two or the three-way analysis of variance (ANOVA) tools of the SIGMA Plot software’s data analysis package. **p < 0.001 and *p < 0.05 were considered significant. Independent experiments were performed at least two times.

## Supplementary Information

**Additional file 1.** Schematic representation of the theoretical model employed to calculate the maximum number of ZnO NCs encapsulated within a single EV and of TNH and TNH^CD20^ assembly procedures; EDS maps of EVs; fluorescence microscopy images and colocalization experiment of the TNH.

## Data Availability

All data generated or analysed during this study are included in this published article and its Additional file.
