# Supplementary material for: Nanotechnological engineering of extracellular vesicles for the development of actively targeted hybrid nanodevices
Source: Cell Biosci. 2022 May 14;12:61. doi: 10.1186/s13578-022-00784-9 (PMC9107671; doi:10.1186/s13578-022-00784-9)
Supplement: Supplementary file 1 — Additional file 1. Schematic representation of the theoretical model employed to calculate the maximum number of ZnO NCs encapsulated within a single EV and of TNH and TNHCD20 assembly procedures; EDS maps of EVs; fluorescence microscopy images and colocalization experiment of the TNH. [file 13578_2022_784_MOESM1_ESM.docx]

**Supporting Information**

**Nanotechnological engineering of extracellular vesicles for the development of actively targeted hybrid nanodevices**


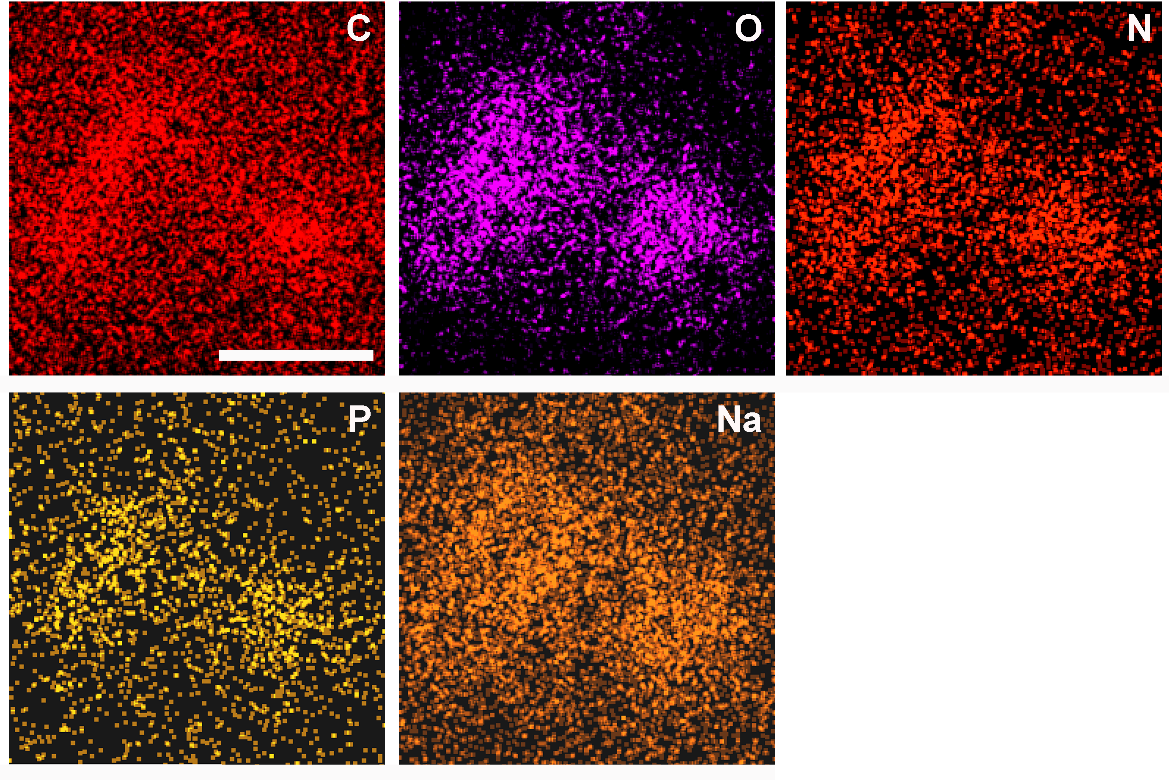


**Figure S-1** EDS elemental maps of carbon (C), oxygen (O), nitrogen (N), phosphorus (P) and sodium (Na) related to BF-TEM micrograph of Fig 2b. Scale bars: 200 nm.


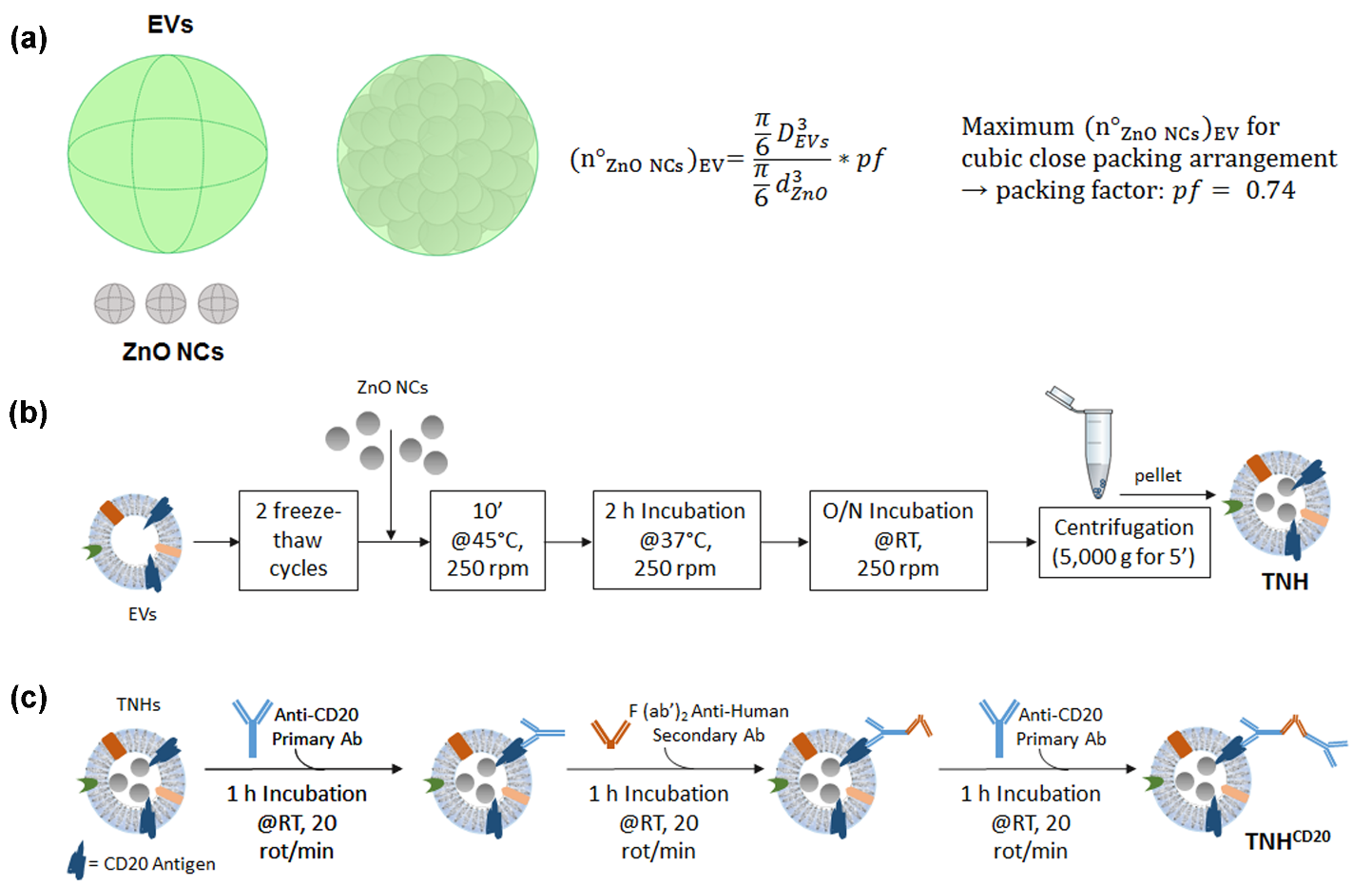


**Figure S-2** Schematic representations of: **a** the theoretical model employed to calculate the maximum number of ZnO NCs encapsulated within a single EV; **b** TNH assembly procedure, combining amino-functionalized ZnO NCs and EVs extracted from B lymphocytes; **c** preparation of TNH^CD20^, conjugating the TNH with anti-CD20 targeting ligands.


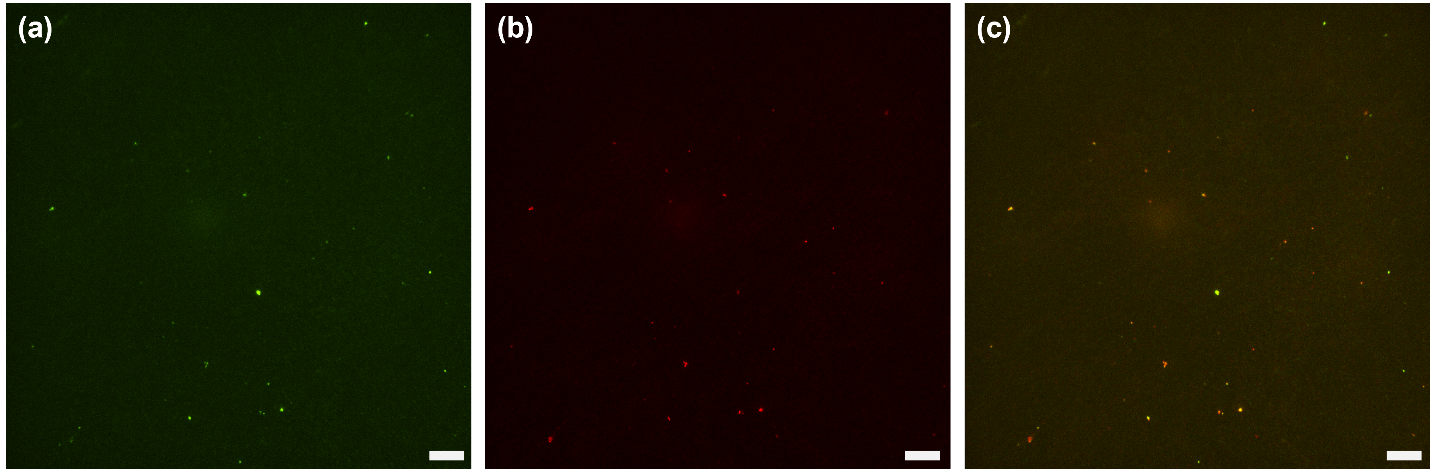


**Figure S-3** Example of TNHs fluorescence microscopy colocalization images. **a** EVs labelled with Wheat Germ Agglutinin (WGA) conjugated with Alexa Fluor 488, **b** ZnO NCs labelled with Atto 550-NHS ester and **c** merge of the two image which allows to assess the assembly of TNHs nanoconstructs as colocalized yellow spots. Scale bars: 10 µm.





**Figure S-4.** NTA measurements of EVs-CD20 and the relative control sample (EVs) obtained without the addition of the primary antibody were analyzed. Specifically, the two samples were measured at the end of each incubation step, i.e. after the addition of: (i) first Rituximab (top panel), (ii) secondary antibody F(ab’)_2_ fragment (middle panel) and (iii) second Rituximab (bottom panel). The three measurements allowed to monitor the evolution of the hydrodynamic size of the samples during the functionalization process, showing no evident aggregation phenomena.
